# Editorial Comment to Prominent response to platinum‐based chemotherapy in a patient with *BRCA2* mutant‐neuroendocrine prostate cancer and *MDM2* amplification

**DOI:** 10.1002/iju5.12291

**Published:** 2021-05-04

**Authors:** Takashi Matsumoto, Masaki Shiota

**Affiliations:** ^1^ Department of Urology Graduate School of Medical Sciences Kyushu University Fukuoka Japan

In this issue, Daimon *et al*.^1^ reported on the efficacy of platinum‐based chemotherapy in patients with neuroendocrine prostate cancer harboring *BRCA2* mutations. Docetaxel administered after progression to castration‐resistant prostate cancer (CRPC) initially suppressed disease progression, but prostate‐specific antigen (PSA) levels remained constantly elevated during the subsequent administration of enzalutamide and cabazitaxel. Eventually, the patient was pathologically diagnosed as adenocarcinoma with neuroendocrine differentiation. After *BRCA2* mutation with *MDM2* amplification and loss of heterozygosity of *RB1* were identified by targeted next‐generation sequencing, the patient is currently undergoing platinum‐based chemotherapy, decreasing PSA level.

The patient was diagnosed with prostate cancer with a high Gleason score at the relatively young age of 55 years with no family history, although it is unknown whether this patient has germline or somatic mutation in *BRCA2* gene. Because *BRCA2* is essential to maintain genomic integrity by homologous recombination of double‐strand breaks, *BRCA2* mutations accumulate genomic errors and increase the risk of carcinogenesis. Recently, Nyberg *et al*.^2^ reported an increased incidence of prostate cancer among *BRCA2* mutation carriers in a prospective cohort study (EMBRACE trial).

It has been reported that carriers of *BRCA2* mutations respond well to platinum‐based chemotherapy.^3^ Here, the patient carried *BRCA2* mutation (p.N2345Qfs*20), which involves the DNA‐binding domain, and suggested pathogenic by causing the homologous recombination deficiency.^4^ Poly (ADP‐ribose) polymerase (PARP) inhibitor was developed for metastatic CRPC with *BRCA1* or *BRCA2* mutation. PARP inhibitors are expected to be effective in this case. However, there have been no reports on the efficacy of PARP inhibitors in patients treated with platinum drugs. Recently, *BRCA* reversion mutations restoring its function were reported as a mechanism obtaining resistance to PARP inhibitor as well as platinum‐based chemotherapy.^5^ Therefore, subsequent report on this case after the use of PARP inhibitor would be important with sequential genomic analysis on *BRCA2*.

## Conflict of interest

Masaki Shiota has received honoraria from AstraZeneca.
